# A Mixed-Method Study on COVID-19 Prevention in Iranian Restaurants

**DOI:** 10.3389/fpubh.2020.585290

**Published:** 2021-01-25

**Authors:** Fatemeh Mohammadi-Nasrabadi, Yeganeh Salmani, Nasrin Broumandnia, Fatemeh Esfarjani

**Affiliations:** ^1^Food and Nutrition Policy and Planning Research Department, Faculty of Nutrition Sciences and Food Technology, National Nutrition and Food Technology Research Institute, Shahid Beheshti University of Medical Sciences, Tehran, Iran; ^2^Urology and Nephrology Research Center, Shahid Beheshti University of Medical Sciences, Tehran, Iran

**Keywords:** Covid-19 prevention, restaurants, customers, managers, public health

## Abstract

**Background:** In the face of the COVID-19, as a public health emergency, the restaurant industry is struggling to organize itself. The aim of this study is to determine the knowledge, attitude, and practice and also the perceptions of restaurants' customers and managers toward COVID-19 prevention.

**Methods:** This cross-sectional study was conducted using the mixed-method approach. Two online questionnaires were undertaken through WhatsApp Messenger among the 210 customers and 50 managers of restaurants. Multivariate linear regression analysis was conducted to identify the predictors of knowledge, attitude, and practice toward COVID-19 prevention. Then semi-structured, in-depth phone interviews were conducted with 45 subjects to identify their perceptions about the restaurant industry during the COVID-19 pandemic.

**Results:** The majority of customers had moderate knowledge (72.4%), positive attitude (90.5%), and desirable practice (38.6%); whereas the majority of managers had sufficient knowledge (50%), negative attitude (82%), and acceptable practice (58%) toward the prevention of COVID-19 in restaurants. Multiple linear regression analysis showed with increasing each 10 years in the age of the customers, the practice score significantly decreased (Beta = −0.155, *p* < 0.05). Moreover, qualitative results revealed three categories (1. restaurant industry, 2. social media, and 3. government) in 9 themes with 32 sub-themes which were explored based on the perception of the participants toward COVID-19 prevention in restaurants.

**Conclusion:** The majority of restaurant customers and managers have sufficient knowledge and acceptable practice, but a positive attitude among customers and a negative attitude among managers about the prevention of COVID-19 were shown. There is an urgent need to understand public awareness about preventing COVID-19 in restaurants at these critical moments. The results seek to provide strategies for the policymakers and restaurant industry to plan the specific educational intervention about how to manage future crises and public health improvement.

## Introduction

Coronavirus disease (COVID-19) outbreak was declared by the World Health Organization (WHO) on 30 January of 2020 as a Public Health Emergency of International Concern (PHEIC) (1). The virus has rapidly been spreading among people through close contact and often via small droplets produced during coughing, sneezing, or eating together (2, 3). The influence of COVID-19 falls into the actual impact of public health, supply chain, and change in what and where people want to buy their food (4). Since Iran, as one of the top 10 countries that have the highest incidence of infection, and the restaurant environment is one of the places where the disease can spread easily, assessing the level of restaurant customers' and managers' KAP and perceptions about COVID-19 can be an effective step in controlling the disease.

There is no peer-reviewed literature examining the COVID-19's ability to stay infectious on foods (5). According to the WHO reports, food contamination can occur through hands, sneezing, and coughing of workers, so proper handling is especially important if the airborne droplets carrying the virus land on ready-to-eat foods. COVID-19 is not a foodborne virus and cannot survive or thrive in food; however, the virus spreads through human contact, and cooking food at the right temperatures can inactivate the virus (1, 6, 7). Therefore, high-temperature heating of the food, preferably over 70°C that inactivates the coronavirus is very important. It has been found that the virus remains stable even at −20°C or less (8).

Moreover, the use of personal protective equipment is crucial to reducing COVID-19 viruses' transmission. Gowns and gloves are recommended as a contact precaution, and masks are recommended as a droplet precaution. It is advised to minimize the contact between people during the outbreak; therefore, online food deliveries are more desirable. These allow physical distancing between customers and sales personnel (9). However, effective infection prevention and control practices depend on the workers' awareness. A poor level of knowledge has been implicated in the rapid spread of the infection after the reopening of restaurants (10).

During the COVID-19 pandemic, widespread customers mistrust the foodservice industry, and eating out or even ordering food from their homes has been created (6). What needs to be done is a study on how the customers react in the post-COVID-19 world. As of now, it is important to make them aware that COVID-19 is not a food-borne virus. The aim of the present was to determine the knowledge, attitude, and practice of participants to reach their views for restaurant managing during the COVID-19 pandemic in order to plan the educational intervention for the target groups of restaurants' staff and the customers.

## Materials and Methods

### Study Design, Population, and Data Collection

This cross-sectional study was conducted by using the mixed method approach among restaurant managers and customers (aged 20 years and above) in three phases.

### Phase I

Eighty-seven restaurants from five districts (North, East, West, South, and Center) of Tehran, the capital city of Iran, were selected randomly based on the total number of restaurants in the city. The online questionnaires' link was sent to the restaurant managers to complete their questionnaire. Fifty of the managers filled out the form. Due to the fact that all the districts do not have an equal number of restaurants, a proportion to size approach has been used to select restaurants per district to reflect the variation in the number of restaurants in each district.

Online managers' questionnaire with a total of 91 scores:

- Four socio-demographic questions included age, gender, literacy, and source of information.
- Six knowledge questions with a score range of 0–6, with “True,” “False,” and “I Don't Know” answers. Each correct response weights 1 point and 0 for incorrect responses and “I don't know” which classifies scores as *low (0–2), moderate (2-4), and good (4-6)*.
- Five attitudes related 4 item Likert questions with the responses of *disagree, probably, agree*, and *strongly agree*. Each weighing 1–4 scores, respectively, which classify the scores as *strongly negative (*<*10), negative (10-15), positive (15-18), and strongly positive (18-20)*. Some questions were reversed to diminish the biases of giving a single similar response in all the items.
- Thirteen questions about the health and food safety practice of restaurant managers. Five Likert-item questions with the responses of *never, rarely, sometimes, often*, and *always*, each weighing 1–5 scores, respectively, which are classified as *weak (*<*30), acceptable (30–60), desirable (60–65)*.
- A single question about their need for education and how they can increase their knowledge in this subject.

### Phase II

Each manager should send the second online questionnaires' link for their 10 randomly selected customers from a list of permanent customers with access to What App messenger to provide every permanent customer in each of the restaurants equal opportunity of being selected for participation. Out of the 500 customers, 253 (50.6%) responded; however, 210 (42%) of the completed questionnaires were eligible to participate in the study.

Online customers' questionnaire with a total of 45 scores:

- Five socio-demographic questions that including age, gender, occupation, literacy, and source of information.
- Eleven knowledge questions with a score range of 0–11 with “True,” “False,” and “I don't know” answers. Each correct response weights 1 point and 0 for incorrect responses and “I don't know,” which classifies scores as *low (0–5), moderate (5-10), good (10,11)*.
- Six attitude questions related 4 items Likert with the responses of *disagree, probably, agree* and *strongly agree*, each weighing 1–4 scores, respectively, which classifies the scores as *strongly negative (*<*10), negative (10-15), positive (15-20), and strongly positive (20-24)*.
- Two Five Likert-item questions about the health and food safety practice with the responses of *never, rarely, sometimes, often*, and *always*, each weighing 1–5 scores, respectively. The questions were classified as *weak (0–2), acceptable (3-6), desirable (7-10)*.

There were a lockdown and social distancing of all citizens during the data collection. So in order to limit the spread of the disease, we preferred to use an online survey portal. In phase I and II, the survey instrument constituted close-ended questions and took ~15 min to complete during the period of 1–15th September 2020. The questionnaire was divided into three parts including the participants' characteristics, knowledge, attitude, and practice toward COVID-19.

### Phase III

In this phase, a qualitative study was conducted using semi-structured, phone interviews with 45 subjects (15 restaurant managers, and 30 customers randomly selected from phases I and II) who were willing to participate in phone interviews. Open-ended interview questions were developed by the study authors and reviewed by an academic review panel. The question explored the participants' perceptions and demands about restaurants in the COVID-19 pandemic. The subject of the interview was about their views on restaurants, social media, and the duty of government during the COVID-19 pandemic. All of the subjects' explicit permission was sought for audiotaping. Each interview lasted from 15 to 20 min. In order to protect their identity, each participant received a coded number, which was used instead of their actual names during the data analysis. The interviews were conducted by the 3 experts in telephone interview, who had good communication. They stopped the interview once data saturation occurred (11).

The questions were “In your opinion, what are the most important challenges of restaurants' managers and customers during the COVID-19 pandemic, and what do you think about improving the situation of restaurants during these periods?” All interviews were recorded and write then final transcripts were re-read to obtain categories until themes were developed using directed content analysis and constant comparison methods. The results were further checked and confirmed by some of these key informants. All research details including procedures, actions, and decisions were documented for audit purposes (12).

### Validation and Pilot Study

The questions were formulated based on the WHO and CDC guidelines and reports on COVID-19 (13). However, we adapted and modified a previously published tool for assessment of Knowledge, Attitude, and Practice (KAP) toward the prevention of respiratory tract infections (6, 14–18). Then, questions inquiring about the attitude toward preventive measures were modified to reflect the attitude and not actual practice. The preliminary phase was conducted to assess the validity and reliability of the questionnaire before using it. Initially, three experts in the field of epidemiology and nutrition and food researchers at Shahid Beheshti University of Medical Sciences were asked to evaluate the questions. Finally, six items on which disagreement among the experts was reported were removed from the final version.

Pretesting was the next step in which the last version of the questionnaire was completed by five managers and 20 customers who were excluded later from the study sample. They were asked to fill out the questionnaire twice 2 weeks apart. The collected data were used to assess internal consistency using Cronbach's alpha, as well as test-retest reliability using the intra-class correlation coefficient. The results showed adequate internal consistency and reliability (with Cronbach's alpha = 0.72 and intra-class correlation coefficient 0.96). The final questionnaire was piloted with three participates, and their comments regarding the clarity of the questions were sought. No further item was removed from the final version.

### Ethical Approval

This study was approved by the Ethics Committee of National Nutrition & Food Technology Research Institute (NNFTRI), Shahid Beheshti University of Medical Sciences, Tehran, Iran (Grant No. IR.SBMU.RETECH.REC.1399.125). All respondents were guaranteed anonymity, and they provided informed consent.

### Statistical Analysis

Fully completed questionnaires were extracted from Google Forms and exported to a Microsoft Excel 2016 for cleaning and coding. The cleaned data were exported to the SPSS software ver. 22. Numerical and categorical data were summarized as frequencies. Chi-square and ANOVA tests were used to determine the relationship between knowledge and attitude scores and socio-demographic variables. In the case of a significant ANOVA test, *post-hoc* analysis (LSD) was performed for multiple comparisons between each category. Multivariate linear regression analysis was conducted by using the socio-demographic variables as independent variables, and knowledge, attitude, and practice scores as the outcome variable to identify the predictors of knowledge, attitude, and practice toward the COVID-19 prevention.

After each interview of phase III, the notes were organized based on the interviews' question. All the records were transcribed verbatim and compared to the notes to fix potential discrepancies. The final transcripts were read repeatedly to achieve immersion and obtain a sense of the whole as one would read a novel. All the data relevant to each category were identified and examined using the constant comparison method (19). The emerged categories were used to organize and group the codes into meaningful themes.

## Results

The majority of customers were women (79.5%) and the majority of managers were men (n = 42, 84%). About 60% of the customers were under 50 years of age and 58% of the managers were 40 years old and above. Overall, 88% of the customers and 92% of the managers had a bachelor's degree and above. About half of the customers (59%) were employed. The most important source of information of all participants on COVID−19 was social media (46%) (Table 1).

**Table 1:** Socio-demographic characteristics of the participants (*n* = 260).

**Variables**	**Customers *N* = 210**	**%**	**Restaurant managers *N* = 50**	**%**
**Gender**				
Male	43	20.5	42	84
Female	167	79.5	8	16
**Age (year)**				
20–30	33	15.8	4	8
30–40	48	22.9	17	34
40–50	41	19.5	15	30
50–60	57	27.1	10	20
60 ≤	31	14.8	4	8
**Education**				
≤ Diploma	25	12	4	8
BSc degree	82	39	19	38
MSc degree	57	27.1	24	48
PhD	46	21.9	3	6
**Occupation**				
Unemployed	11	5.2	–	–
Housewife/houseman	41	19.5	–	–
Retired	35	16.7	–	–
Employed	123	58.6	50	100
**Source of information on COVID-19**				
Social media (WhatsApp, Instagram, Telegram)	96	45.7	20	40
Family/friends	9	4.3	23	46
TV and radio	59	28.1	5	10
Others^*^	46	21.9	2	4

* * Others: WHO website/CDC website/Local rules/scientific articles*.

**Figure 1:**
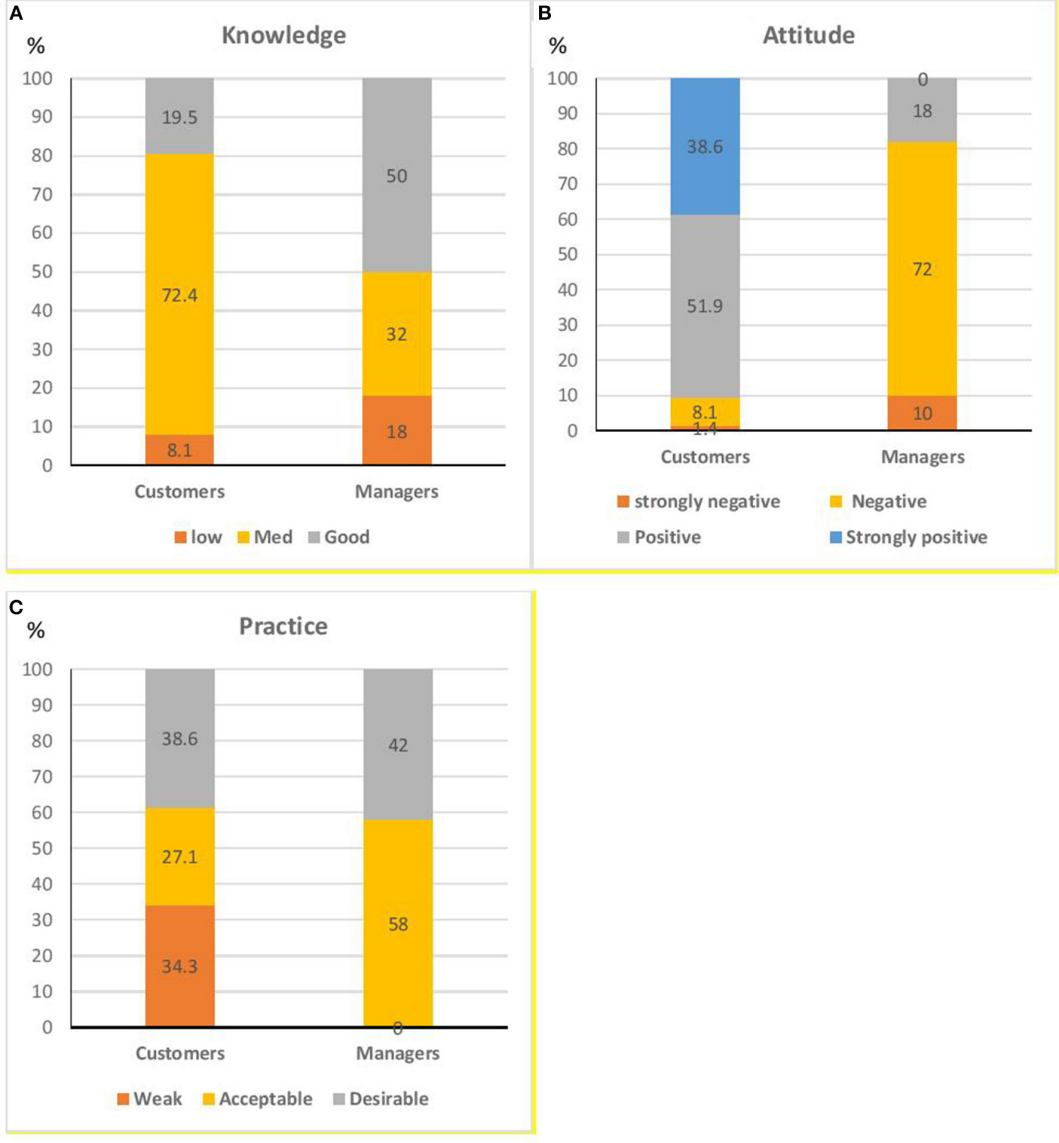
Relative frequency of different degrees of **(A)** Knowledge, **(B)** Attitude, and **(C)** Practice of restaurants' consumers and managers toward Covid-19 prevention.

Figure 1 showed the frequency (%) of knowledge (low, moderate, and good), attitude (strongly positive, positive, negative, strongly negative), and practice (weak, acceptable, desirable) of restaurants' consumers and managers toward Covid-19 prevention. The majority of customers had moderate knowledge (72.4%), positive attitude (90.5%), and desirable practice (38.6%); whereas the majority of managers had sufficient knowledge (50%), negative attitude (82%), and acceptable practice (58%) toward the prevention of COVID-19 in restaurants. Also, 82% of them felt the urgent need for further virtual educational training in this matter.

**Figure 2:**
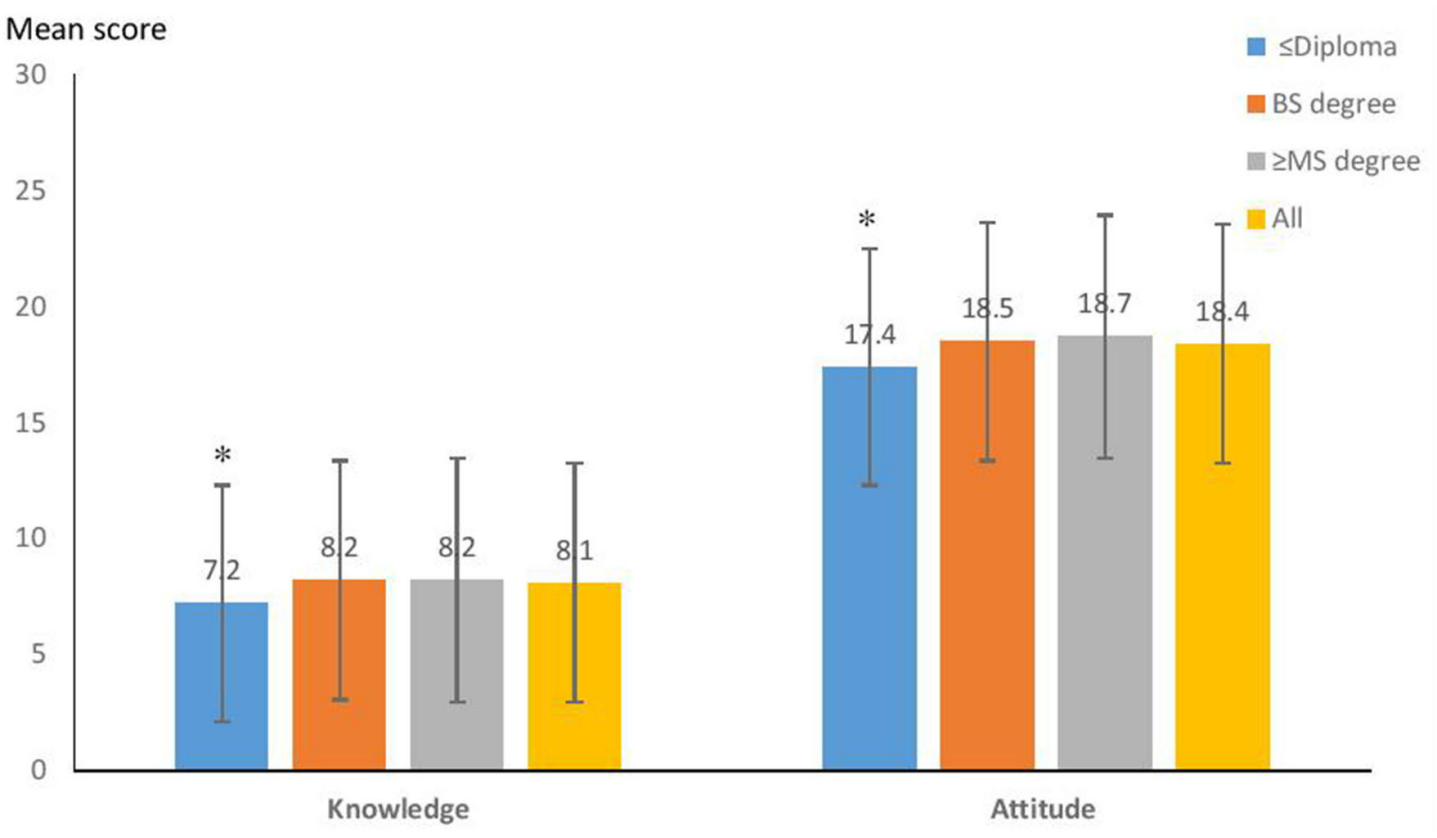
Relation of the educational level of the customers with their knowledge and attitude scores toward the COVID-19 prevention (*n* = 210). *Statistically significant difference with other groups by ANOVA test at *P* < 0.05; Practice score was not significantly related to educational level.

The result of the ANOVA test showed that the mean of knowledge and attitude of customers was significantly different by educational level (Figure 2). The relation between education and age of the customers and their practice scores was significant, too (*P* < 0.05); however, the relation of other socio-demographic characteristics (gender, job, and source of information) was not significant. On the other hand, there was no statically significant difference in the level of knowledge and attitude scores of the managers toward COVID-19 with the socio-demographic variables. Only the practice score of the managers was significantly related to their source of information according to the Chi-square test results (*p* < 0.05) (Table 2).

**Table 2:** Relation between the customers' age, educational level, and the managers' source of information and their practice score toward COVID-19 prevention.

**Variables**	**Practice score**			**Total**
	**Weak**	**Acceptable**	**Desirable**	
Education^*^			
≤ Diploma	7 (28)	15 (60)	3 (12)	25 (100)
BS degree	28 (34.1)	23 (28)	31 (37.8)	82 (100)
≥ MS degree	37 (35.9)	19 (18.5)	47 (45.6)	103 (100)
Age^*^			
20–30	13 (39.7)	3 (9.1)	17 (51.5)	33 (100)
30–40	15 (31.3)	1 (2.1)	32 (66.7)	48 (100)
40–50	21 (51.2)	6 (14.6)	14 (34.1)	41 (100)
50–60	12 (21.1)	30 (52.6)	15 (26.3)	57 (100)
60 ≤	11 (35.5)	17 (54.8)	3 (9.7)	31 (100)
Total	72 (34.3)	57 (27.1)	81 (38.6)	210 (100)
Source of information^**^			
Social media	0	10 (50)	10 (50)	20 (100)
Family & friends	0	17 (73.9)	6 (26.1)	23 (100)
TV & radio	0	0	50 (100)	5 (100)
Others^†^	0	2 (100)	0	2 (100)
Total	0	29 (58)	21 (42)	50 (100)

* *Statistically significant by χ^2^ at P < 0.05. Other socio-demographic characteristic (sex, job, and the source of information) was not significantly associated with consumers' practice scores*.

** *The practice score of managers was significantly related to their source of information by χ^2^ at P < 0.05. Other socio-demographic characteristic (age, education, sex, and job) was not significantly associated with managers' practice scores*.

† *Others: WHO website/CDC website/Local rules/scientific articles*.

The multiple linear regression analysis results revealed that the main socio-economic positive predictor of knowledge and attitude toward COVID-19 prevention was the educational level of customers, whereas age was the major negative predictor of practice in them. In other word, with increasing each 10 years in the age of the customers, the practice score significantly decreased (Beta = −0.155, *p* < 0.05) (Table 3).

**Table 3:** Socio-economic predictors of knowledge, attitude and practice of customers toward the COVID-19 prevention.

**Relation to socio-economic predictors**	**B**	**Standard error**	**Beta**	***P*-value**
Knowledge	Education	0.273	0.127	0.168	0.032^*^
	Job	0.074	0.128	0.044	0.567
	Gender	−0.272	0.273	−0.069	0.319
	Age	−0.010	0.084	−0.009	0.902
Attitude	Education	0.490	0.235	0.164	0.039^*^
	Job	−0.092	0.238	−0.030	0.699
	Gender	−0.180	0.507	−0.025	0.722
	Age	0.221	0.156	0.099	0.158
Practice	Education	0.050	0.068	0.057	0.466
	Job	−0.004	0.069	−0.005	0.949
	Gender	−0.107	0.147	−0.051	0.468
	Age	−0.101	0.045	−0.155	0.028^*^

* *Statistically significant by multiple linear regression analysis at P < 0.05*.

The qualitative result showed that the perceptions of customers and managers about COVID-19 prevention in restaurants are classified into three categories in 9 themes with 32 sub-themes (Table 4). Observance of all hygiene and sanitizing principles and guidelines completely, social distancing in restaurants, was the most frequent suggestion of the restaurant customers. Also, the majority of customers' prefer was showing all steps of food preparation on social media online. On other hand, all the restaurant managers asked the government for free tax during this time and the majority of them want the government to support COVID-19 testing for staff. They also suggested that the government hold some continuing educational webinars for restaurants' staff.

**Table 4:** The most important concepts extracted from consumers and managers perceptions on the restaurant industry during COVID-19 pandemic.

**Category**	**Theme**	**Sub-theme**	
		**Customers**	**Restaurant manager**
Restaurant industry	Personal Hygiene	• Observance of complete hygiene and principles, according to the protocols for staffs • Restaurants should open with a half of the restaurants' capacity for social distance	• Observing the constant hygiene of each staff and customer • Maintaining environmental hygiene • Observance of 2 meters distance in the restaurants
	Environment hygiene	• Disinfect tables and chairs • Sanitizing toilet	• Disinfect all equipment • Digital payment
	Food hygiene	• Observing food hygiene specially in raw food • Serving the meal in the heaters on the tables • Keeping the plates, spoons, and forks warm on the tables	• Serving well-cooked foods • Using disposable and packaged containers
	Delivering	• Online food deliveries are more desirable • Observing the personal hygiene for delivery drivers • Restaurants should insert the label of reheating on the food packages	• Disinfecting the final package before delivery
Social media	Advantages	• Restaurants can show all the steps of food preparation on the website	• Keep posting information on social media platforms
	Disadvantage	• Infodemic and disinformation • Fake advertisements	• Disinformation • Unreal advertisements against restaurants
Government	Restaurant inspection	• Continuous online monitoring by Ministry of Health and Medical Education • Quick handling of consumer complaints	• Serious audit and inspection by the Union. • Correct and strict screening
	Funding	• The government should provide funding for restaurants being equipped	• Free tax during this time • Support COVID-19 testing for staffs
	Education	• Sending educational health messages	• Holding educational webinars

## Discussion

Since it is not possible to work remotely in the food industry and employees have to continue working in the former work environment, the food production chain (supply, preparation to cook, and delivery) must be operated and controlled under certain health conditions (20).

To the best of our knowledge, this is the first mixed-method study toward COVID-19 among restaurants. In this study, the majority of participants were well-educated, and overall, the rate of correct answers for the knowledge questionnaire about COVID-19 prevention in the restaurants was sufficient.

Educational status was the most important factor in improving knowledge and attitude in both groups so that with increasing the educational level, the score of knowledge was increased toward the prevention of COVID-19.

Similarly, a study in China (2020) well-confirmed revealed that there was a significant relationship between the Chinese residents' education level and the level of their knowledge about COVID-19 (21).

Since the majority of customers were < 50 years of age and well-educated and familiar with social media, this platform was the most important source of information among them toward COVID-19. Having sufficient knowledge may reflect the successful distribution of information about COVID-19 by different media. The widespread use of the Internet and its availability to wider sectors of society has made it a major source of information for the public in using this information source. Similar to our findings, other studies reported that the participants usually obtained their information about infectious diseases through the Internet and watching TV (22–24).

Another study showed that the media plays a significant role in making the public aware of hygiene measures specified by health authorities to help the pandemic. Together with customer demands for safe food, they have engendered changes in food production practices. Customers' in?uence safety practices by purchasing only from places they feel are safe, which they judge from the delivery experience, the packaging specifics, and the end product. Social media posts about restaurants' hygiene and sanitization procedures can be used strategically to develop transparency from the restaurant to the customer, as they show how the restaurant has adopted safety criteria (25).

A cross-sectional study in Bangladesh in 2020 aiming to raise awareness and attitudes among the different sections of society about COVID-19, showed that the participants' most important source of information about the COVID-19 disease was social media (26). Nowadays, social media, in addition to being the primary source of information, is also a vehicle for news and events. During a crisis like the COVID-19 pandemic, social media should be mastered and employed responsibly. It seems that due to the important situation of this epidemic disease and given the sensitivity of the people to it, they would actively learn the knowledge of COVID-19 from various sources of information. The significant positive association between levels of education and COVID-19 knowledge shows these findings.

In the present study, although both the customers and managers had sufficient knowledge and acceptable practice, the customers' attitude was positive but the managers' attitude was negative. It seems that many factors could affect the negative attitude of them. One of the important factors can be the locking down of the restaurants during this time and another factor is, after the reopening permission of the restaurants, the customers were afraid of going to eat there or even order food to be sent to their home because they were not uncertain about the restaurants' condition with regard to spreading COVID-19. Finally, economic problems that the restaurant industry suffered during this period have a negative impact on the attitude of restaurant managers, probably because they are responsible for their staffs and customers, and also they were responsible for paying employees' salary in case of illness (COVID-19 Positive) with the limited support of the government.

Based on the customers' important views there would be more confidence among them in going to or ordering food from the restaurants, if the restaurants observe all the hygiene and sanitizing principles as well as the protocols issued by the health authorities, including wearing a mask by the staffs, performing social distancing. A study in China in 2020 indicated that observing personal hygiene by staff involved in food handling was of great importance to all food-related centers. COVID-19 positive people should not be allowed to be present in food-related environments. The COVID-19 disease can be asymptomatic in the early stages, meaning that asymptomatic staffs may actively transmit the virus to food and food-handling surfaces (14).

Another view was that they should only deliver food with online ordering and they should insert the label of reheating on the food packages.

Also, the customers suggested online monitoring is required by the Ministry of Health and Medical Education. It seems that, reminding the customers that all the principles based on the standard health protocols have been met in social media can increase the customers' trust during the COVID-19 pandemic.

Restaurant managers believed that serious inspections are required by the Union. In addition, the majority of restaurant managers expected the government to provide virtual training for the restaurant staff. However, as the results showed, their attitude toward COVID-19 has been negative and it seems that they need specific training courses for updating their information in this regard. The findings of a study in China in 2020 showed that advanced training courses and special attention could lead to raising knowledge and attitude (27).

To promote and develop the equipment and sustain safety steps (such as digital menus, digital thermometers, no-touch-hand sanitizer, hotboxes for delivering, using robots instead of waitress, and automatic serving), governmental financial support is needed.

The managers believed that the government should support COVID-19 testing for the restaurant staff. It seems that, due to the high price of testing, they are not inclined to do such tests, until symptoms appear.

Most managers appear resistant to make changes to the way of cooking, packaging, delivery, which could be due to the economic shock for this industry. Economic fears have negative impact on managers' creativity and innovation at this time. The restaurant industry will have to re-create itself as an appropriate strategy for surviving in this time and post COVID days.

Totally, the restaurant industry should prepare for future crises. The managers must follow all instructions issued by the authorities and that they have to reduce workplace risk to the lowest reasonably practicable level by taking preventative measures. Responsible managers will join with their governments' fight against COVID-19 by working with their sector bodies to protect their workers and the customers. Additionally, inspectors should carry out compliance checks nationwide to ensure that employers are taking the necessary steps.

Based on the United Kingdom's COVID-19 secure guidance for managers and staff, the managers should make sure that the risk assessment for their business addresses the risks of COVID-19, and the employers and workers should always come together to resolve issues (28).

The National Academy of Sciences defines resilience as “the ability to plan and prepare for, absorb, recover from, and adapt to adverse events.” Creating these four sections that can provide insights into how restaurants are responding to the pandemic in terms of food safety and the public health involved, workers, and customers (29).

Also, the governments should support the restaurant managers to increase their staffs' knowledge and attitude by online intensive update courses that can lead to desirable practice for the prevention of COVID-19 in the community. Therefore, the results of this study can be useful for the restaurant industry to create new ways and planning for future educational intervention to help for breaking the cycle of COVID-19 in the communities.

## Limitations

The main limitation is the speed at which general knowledge both changes and grows in relation to what the general public knows about the transmission of COVID-19 which has changed the context in which the survey work was done compared to today. Due to the long duration of the COVID-19 pandemic, the majority of the restaurants were closed and the most of managers were not willing to cooperate with this study, so the sample size of managers was limited. Moreover, the response rate of 50% also limits the generalizability of the result.

## Conclusion

The majority of restaurant customers and managers have sufficient knowledge and acceptable practice, but positive attitude among customers and negative attitude among managers about the prevention of COVID-19 was shown.

Social media has the main role and responsibility in increasing the community's information during the COVID-19 crisis. The authorities should provide a guideline for the restaurants for the prevention of the COVID-19 disease. It is an urgent need for training programs to improve the understanding of the risks and prevention strategies among the restaurant managers and public awareness and also specific educational intervention by the government and stakeholders for crises and public health improvement.

## Data Availability Statement

The original contributions presented in the study are included in the article/supplementary material, further inquiries can be directed to the corresponding author/s.

## Ethics Statement

This study was approved by the Ethics Committee of National Nutrition & Food Technology Research Institute (NNFTRI), Shahid Beheshti University of Medical Sciences, Tehran, Iran (Grant No. IR.SBMU.RETECH.REC.1399.125). All respondents were guaranteed anonymity, and they provided informed consent.

## Author Contributions

FE: concept, design, statistical analysis, and supervision. YS: acquisition, analysis, and interpretation of data. FE, YS, and FM-N: drafting of the manuscript. FM-N and NB: critical revision of the manuscript. All authors contributed to the article and approved the submitted version.

## Conflict of Interest

The authors declare that the research was conducted in the absence of any commercial or financial relationships that could be construed as a potential conflict of interest.
